# An Evaluation of Motion Trackers with Virtual Reality Sensor Technology in Comparison to a Marker-Based Motion Capture System Based on Joint Angles for Ergonomic Risk Assessment

**DOI:** 10.3390/s21093145

**Published:** 2021-05-01

**Authors:** Jan P. Vox, Anika Weber, Karen Insa Wolf, Krzysztof Izdebski, Thomas Schüler, Peter König, Frank Wallhoff, Daniel Friemert

**Affiliations:** 1 Institut of Technical Assistance Systems (ITAS), Jade University of Applied Sciences, Ofener Str. 16/19, 26121 Oldenburg, Germany; frank.wallhoff@jade-hs.de; 2 Division Hearing, Speech and Audio Technology HSA, Fraunhofer Institute for Digital Media Technology IDMT, Marie-Curie-Str. 2, 26129 Oldenburg, Germany; insa.wolf@idmt.fraunhofer.de; 3 Department of Mathematics and Technology, University of Applied Sciences Koblenz, Joseph-Rovan-Allee 2, 53424 Remagen, Germany; weber4@hs-koblenz.de (A.W.); friemert@hs-koblenz.de (D.F.); 4 Sport and Exercise Science Research Centre, School of Applied Sciences, London South Bank University, 103 Borough Road, London SE1 0AA, UK; 5 Halocline GmbH & Co. KG, Netter Platz 3, 49090 Osnabrück, Germany; kizdebski@halocline.io (K.I.); tschueler@halocline.io (T.S.); 6 Institute of Cognitive Science, University of Osnabrück, Wachsbleiche 27, 49090 Osnabrück, Germany; peter.koenig@uni-osnabrueck.de; 7 Institute of Neurophysiology und Pathophysiology, University Medical Center Hamburg-Eppendorf, 20246 Hamburg, Germany

**Keywords:** Qualisys Oqus, HTC Vive, accuracy, virtual reality, lighthouse technology, system comparison, joint angles, motion capture, ergonomic risk assessment

## Abstract

The reproduction and simulation of workplaces, and the analysis of body postures during work processes, are parts of ergonomic risk assessments. A commercial virtual reality (VR) system offers the possibility to model complex work scenarios as virtual mock-ups and to evaluate their ergonomic designs by analyzing motion behavior while performing work processes. In this study a VR tracking sensor system (HTC Vive tracker) combined with an inverse kinematic model (Final IK) was compared with a marker-based optical motion capture system (Qualisys). Marker-based optical motion capture systems are considered the gold standard for motion analysis. Therefore, Qualisys was used as the ground truth in this study. The research question to be answered was how accurately the HTC Vive System combined with Final IK can measure joint angles used for ergonomic evaluation. Twenty-six subjects were observed simultaneously with both tracking systems while performing 20 defined movements. Sixteen joint angles were analyzed. Joint angle deviations between $\pm 6^{\circ }$ and $\pm 42^{\circ }$ were identified. These high deviations must be considered in ergonomic risk assessments when using a VR system. The results show that commercial low-budget tracking systems have the potential to map joint angles. Nevertheless, substantial weaknesses and inaccuracies in some body regions must be taken into account. Recommendations are provided to improve tracking accuracy and avoid systematic errors.

## 1. Introduction

Workplace design and ergonomic construction play very important roles in reducing work-related musculoskeletal disorders that can be caused by forced postures during work [1,2]. In order to prevent work-related musculoskeletal disorders, it is necessary to evaluate workplaces ergonomically [3]. In one common approach, an ergonomics expert observes the employee performing the movements necessary for the planned work process at his/her workplace [4]. Observed postures and the dynamic ranges of movement are incorporated into a risk assessment. Specific joint angles are the basis for these kinds of assessments, e.g., the Rapid Upper Limb Assessment (RULA) [5]. Therefore, we focus on the evaluation of joint angles. If an ergonomic review determines that workers must work in unergonomic postures, the existing workstation must be adjusted.

**Figure 1:**
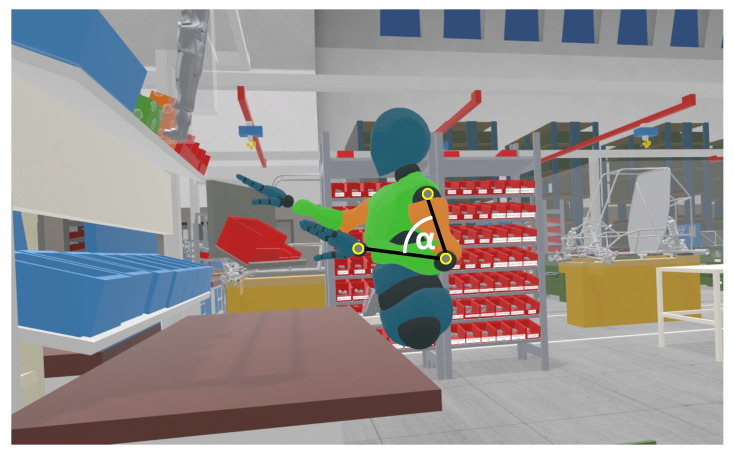
An avatar which performs movements at a virtual workplace. An exemplary representation of the relevant joint positions for the determination of the joint angle of the elbow flexion.

Physical mock-ups are often used to ergonomically adapt an existing workstation. However, this is very time-consuming and means physically redesigning the mock-up. To make this process more efficient, virtual environments can be used. The virtual mock-up can easily be modified, allowing for quick adaptations of the workplace design [6]. Other advantages of virtual mock-ups include higher flexibility and independence of physical space. Furthermore, it is possible to design ergonomic workplaces during the design phase of, e.g., new factories. The goals of integrating virtual reality (VR) for ergonomic assessments are to facilitate the design process, increase design efficiency and reduce costs. An example of a virtual workstation and an avatar representing the user in a motion scenario can be seen in Figure 1.

VR technology also offers the possibility to track body postures based on specific tracker positions (head-mounted-display (HMD), controller and additional tracker). VR systems which allow interactions of the user in VR need this kind of motion capturing to determine a model of the user’s body in relation to the VR environment. For our study, we have chosen the HTC Vive VR System (www.vive.com, accessed on 20 January 2021).). Evaluations of the spatial accuracy of the tracker positions showed good results (Section 2). Therefore, VR technology seems to be a promising tool for ergonomic assessments. For this purpose it is necessary to derive joint angles based on kinematic data (i.e., positions and quaternions of the respective body segments). The inverse kinematic (IK) model is applied to derive such kinematic data using sensor information of the trackers. An exemplary representation of the joint angle of elbow flexion is shown in Figure 1. According to current research, no study has evaluated a VR motion capture system combined with the inverse kinematic model in terms of joint angles of human bodies in motion, which would be relevant for ergonomic motion evaluation. Furthermore, no studies are known of that have investigated the joint angle accuracy of HTC Vive combined with Final IK. In the present paper, joint angles based on a HTC Vive VR tracking system in combination with Final IK (www.root-motion.com, accessed on 20 January 2021) are compared with results from a gold standard marker-based system, Qualisys (www.qualisys.com, accessed on 20 January 2021), to assess the accuracy of the joint angles. Sixteen joint angles were selected which are mainly used in the common motion evaluation schemes Rapid Upper Limb Assessment (RULA) [5] and Rapid Entire Body Assessment (REBA) [7]. When using the HTV Vive trackers, fewer body segments are captured than with a high-cost motion capturing system, and the remaining segment movements are calculated using IK. As the number of VR trackers used for the IK model has influences on the accuracy of the resulting body positions and orientations, a minimum of three trackpoints and a maximum of nine trackpoints were analyzed.

In summary, this paper examines the following research questions:

1. How accurate is the HTC Vive system combined with Final IK compared to the Qualisys optical marker-based motion capture system in terms of calculated joint angles?
2. How does the number of Vive trackers affect accuracy?
3. Is the joint angle accuracy sufficient enough for ergonomic risk assessments?

To answer these questions, a motion study with 26 subjects was conducted in which kinematic data of 20 dynamic movements per subject were recorded simultaneously with HTC Vive + Final IK (hereinafter “Vive”) and the Qualisys system. Time series of the respective joint angles were compared and the differences between the systems were evaluated. The principle processing steps to derive joint angles starting from kinematic data based on tracker information are described in the paper. Additionally, processing steps were necessary to allow the comparison of the time series of both independent systems (same reference; time synchronicity). Furthermore, identified measurement problems of the Vive system such as measurement dropouts and systematic offsets and their corrections are discussed. The derivation of joint angles related to a moving 3D body or kinematic data is usually specific to the sensor technology and application area. Different definitions exist depending on the purpose and the community. In the area of ergonomic workplace assessment, the proprietary motion analysis tool Winkel Daten Analyse (WIDAAN) (German for “angle data analysis”) is well established [8]. Although there are some differences in the processing steps and the WIDAAN joint angle calculation database is not open-source, WIDAAN was used to check the plausibility of the results in this paper.

The paper is structured as follows: In Section 2 the related work is presented. Section 3 describes the experimental setup and data acquisition of the study. Section 4 describes the processing steps of the calculation of joint angles, the synchronisation of the times series of both systems and the treatment of tracking problems. Section 5 presents the approach to compare systems based on joint angles in detail. The results of the system comparison can be found in Section 6, followed by the discussion in Section 7. The paper is concluded in Section 8.

## 2. Related Work

Several papers have been published which examine the spatial motion tracking precision and accuracy of the Vive’s components in static and dynamic conditions.

In [9], the accuracy of the Vive system was investigated based on positions and orientations in a static condition. The HMD was placed on grid lines (grid point error 1.7 ± 0.9 cm) drawn on the floor and the measured HMD position was compared with the grid position (manual HMD placement error <2 cm). Root mean square error (RMSE) values of <0.08 mm and <0.011${}^{\circ }$ were found. The authors reported the occurrence of tracking loss, which can lead to a decrease in accuracy, and proposed recalibration options for improvement. A similar method was utilized in [10] but using the PhaseSpace camera (positioning error <1 cm) as ground truth instead of grid lines. The mean distance between the true position and the measured position was <1 cm. The authors reported problems with the calibration too. A comparative study between the marker-based Vicon system and Vive was investigated in [11]. The experiment included a robot for the simulation of trunk movements and a study with seven subjects performing trunk movements. The optical markers were attached directly to the Vive trackers and the positions and rotations were compared between the systems. No significant differences were found in the robot simulation. The authors reported a RMSE within 0.68 ± 0.32 cm translationally and 1.64 ± 0.18${}^{\circ }$ rotationally. In the thorax rotation of the participants, no differences between Vive and Vicon were found either. The authors concluded that the Vive tracker can be used to accurately track joint motion for clinical and research data. The authors in [12] evaluated the accuracy from VR sensor technology while tracking the position and orientation of an ultrasound probe dummy in laboratory conditions. They compared a VR tracker to an optical tracking system and evaluated the RMSE. They concluded a high accuracy of tracking with an RMSE< 1 mm. For rotational movements an $RMSE<1^{\circ }$ could be achieved. In [13], the Vive system was compared with a laser tracking system in a static scenario. The authors showed that Vive has an accuracy in the lower millimeter range for the detection of positions. However, systematic deviations in the centimeter range were also found when multiple base stations were used. In addition, a deviation of $0.4^{\circ }$ was detected for rotations.

The accuracy of head kinematics has been evaluated in a dynamic scenario using the intra-class correlations (ICC) [14]. In this study, head movements were compared between the HMD values of the Vive and the marker-based Qualisys system (reference). The authors concluded that the ICC was between 0.9 and 0.99 in most cases, indicating excellent agreement between Vive and Qualisys. Weaker agreement was shown for yaw and pitch movements. In [15], the Vive was examined for pose estimation. In the experiment, the accuracy was tested on the basis of positions in static poses and dynamic movements of the trackers. The authors showed that the accuracy has sub-millimetric precision when the tracker is static. However, the accuracy can decrease from millimeters to 5 cm and even up to 80 cm in dynamic situations, depending on the orientation of the tracker to the base station. The authors in [16] positioned a tracker on a rigid structure and performed controlled rotations and translations. A mean angular error <0.4${}^{\circ }$ and a mean transitional error <3.0 mm were determined. They concluded that the Vive system has the potential for tracking valid and reliable kinematic data. In [17] the authors studied the performance of lighthouse technology, which is also used in the Vive system, for applications in biomechanics and robotics. They mounted a tracker on a robot end-effector and compared the poses of the motion trackers with the poses obtained by the end-effector. It was shown that the accuracy is in the millimeter and sub-degree scales. Considering only data in which no tracking loss occurred (93.4%), the accuracy increased to sub-millimeter for positions and sub-degree for orientations. The feasibility of measuring joint angles on an artificial arm was also investigated. The authors concluded that quantifying joint angles is possible. In [18] the spatial tracking performances of all three Vive components (HMD, controller and tracker) were evaluated by a series of adapted standardized tests from the American Society for Testing and Materials international. The determined accuracy in static cases were given with average errors of of 3 mm and 0.5${}^{\circ }$. A maximum of 45% of data had a positional error of less than 10 mm when moving with an average velocity of 900 mm/s, with up to 34% tracking loss. When reducing the velocity to <135 mm/s, the tracking loss decreased to <0.5% for all tracking devices. The tracker showed the least accuracy, followed by the controller. The best accuracy and least tracking loss was found for the HMD, even for higher velocities.

Overall, the accuracy reported for Vive ranged from sub-millimeter and sub-degree scales to 1 cm and $2^{\circ }$ for static evaluations. In the dynamic conditions the results varied from sub-millimeter to a few centimeters because of methodological differences in the movements, reference tracking, and movement executions. Some studies showed problems in calibration and tracker loss. Mostly, the related work shows assessments of the accuracy of individual trackers using robot movements.

In the present study, however, the Vive system was used on human subjects to capture kinematic data. Therefore, tracking errors due to sensor slippage and occlusion (i.e., when a body part covers the optical connection to the base station) were to be expected. Furthermore, the present study focuses on a validation based on joint angles using the Vive tracker data in combination with Final IK. Thus, the accuracy of the joint angles depends not only on the spatial tracking performance of the trackers, but also on the calculation of the IK. The general study design is similar to that of [19], in which motion capture data from a depth camera and sensor suit were compared to a marker-based system on a joint angle basis. There is no comparable related work that evaluated the HTC Vive system combined with Final IK for joint angle measurements based on human movements. Nevertheless, the reported work provides a high level of insight into the properties of the measurement system and potential issues.

## 3. Experimental Setup and Data Acquisition

### 3.1. Participants

In the present study, 26 young healthy adults (16 males, 10 females) participated with informed consent. They all had normal or corrected vision and were free of neurological and musculoskeletal impairments that might have affected movements or cognitive function. The group consisted of participants between 21 and 27 years of age (mean age: 22.80 ± 1.54; mean height: 1.75 ± 0.07 m; mean mass: 74.92 ± 12.28 kg). The study was approved by the ethics committee of the University of Applied Sciences Koblenz and met all requirements for human experimentation in accordance with the Declaration of Helsinki.

For four of the 26 measured participants, not all datasets were recorded and saved for the specified tracker configurations. Two more were excluded due to continuous position jumps of some tracker data to implausible positions (i.e., elbow on the back; see also Section 7.1). Therefore 20 participants were included in the comparison analysis. In total, 144 min of motion data were analyzed, corresponding to 1,077,099 postures as individual samples.

### 3.2. Measurement Procedure

**Figure 2:**
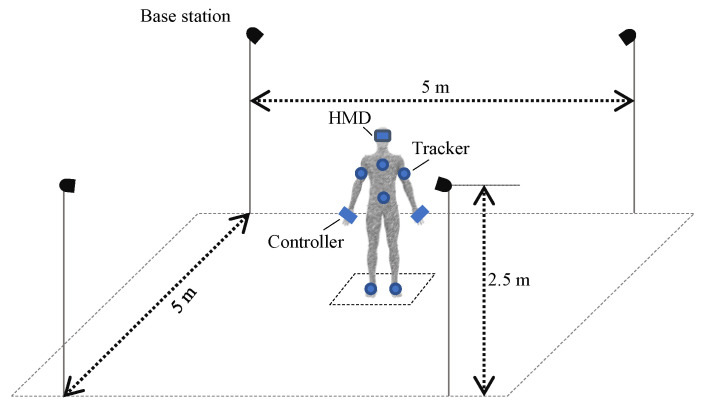
The HTC Vive measurement setup with four base stations and a centrally placed test person with an HMD, two controllers and six trackers.

The participants were equipped with an HMD (HTC Vive Pro), 6 Vive trackers (one at each foot, one at navel height, one at the sternum, one at each upper arm near the elbow) and one Vive controller at each hand. Four Vive Base stations were used to track the Vive devices (HMD, Controller, Tracker). The measurement setup for the HTC Vive system with the four base stations and a participant with an HMD, controllers and trackers is shown in Figure 2.

**Figure 3:**
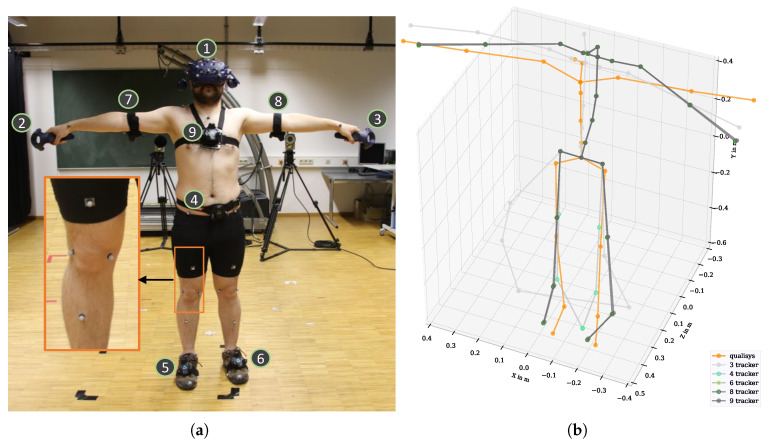
Setup of simultaneous motion measurements with the Qualisys system and the Vive system with different tracker configurations. (**a**) A participant in T-position with attached markers for the Qualisys system and Vive trackers, specified with the numbers 1–9. (**b**) A body skeleton based on the Qualisys system and on different Vive tracker configurations.

The so-called “Lighthouse” technology is based on optical infraredlaser-based distance measurements using trigonometry, supported by an inertial measurement unit (IMU) [13]. The position and orientation of each tracker were determined. At least two base stations are recommended for accurate tracking of positions; however, we used four base stations to avoid tracking errors due to occlusions. The HMD was used to display a virtual environment created with the game engine Unity (version 2018.3.14f1) linked to the VR system. To visualize the participant’s body in VR, an avatar based on Vive tracker data and Final IK (version 1.8) was used. The anthropometric data according to [20] of each subject were collected and entered as measures for the avatar’s size and as input for the calculations in Final IK. Positions and orientations of each participant were then recorded as kinematic data with 90 Hz for five different tracker configurations. The minimal configuration consisted of three trackpoints (head, hand left and right). The trackpoint on the head was built into the HMD. The controller in each hand had another trackpoint. Further trackpoints were on the arms, chest, hip, legs and feet. The kinematic data were stored based on 3, 4, 6, 8 and 9 trackpoints (using trackpoints 1–3, 1–4, 1–6, 1–8 and 1–9, respectively; see Figure 3a). A comparison between the kinematic data obtained from different tracker configurations can be seen in Figure 3b. The movements performed are described in Table 1. The raw Vive kinematic data consist of Cartesian’s positions at the joints of the skeleton and quaternions in a global coordinate system, which were saved in a protobuf format and were then exported to tab separated value (tsv) format.

A marker-based motion capture system (8-camera, Oqus 7/Oqus 5, Qualisys, Gothenburg, Sweden) was used as the gold standard reference system. The reported error of individual marker positions was <1 mm [21,22]. The data were recorded at 100 Hz using a 48-marker model (Qualisys Animation Marker Set). The retroreflective markers were positioned at anatomic landmarks directly on the skin. Qualisys software provides gap filling, calibration and IK to derive finally kinematic data (skeleton data). The Cartesian’s positions at the joints of the skeleton and quaternions were exported in tsv. A fully instrumented participant can be seen in Figure 3a. It is assumed that the resulting joint angles from Qualisys are more accurate, and they were therefore be used as the ground truth angles [23,24].

The subjects were observed simultaneously with both tracking systems while performing 20 defined movements. A second avatar was used to to demonstrate the specific movements of the participant. In the beginning, a synchronization movement was performed: the subject stood in a T-pose, bending his arms $90^{\circ }$ and then stretching them again. This was repeated three times. Before each of the 20 movements and the repetitions, the subject briefly stood in a neutral position. Each movement had to be performed at normal speed with the largest possible range of motion (RoM) and held briefly in the end position. Three repetitions of each movement were performed. Each movement refers to a specific body region. From these, 16 angles were selected and examined using joint angle differences between HTC Vive + Final IK and Qualisys. Descriptions of movements and assignments to the specific joint angles are shown in Table 1. All movement phases were additionally recorded on video.

## 4. Data Processing

Both systems provide kinematic data (positions and quaternions of the respective joints) as time series. Based on the kinematic data, 16 joint angles were calculated for each system. The data were manually annotated and time segments were assigned labels with the respective motion.

For the comparison of the joint angles based on both time series, different additional processing steps were necessary to provide the same reference and synchronicity. A direct implementation of a synchronized data recording was not possible for the commercial systems. Additionally, data drop outs and systematic effects (Section 4.7 and Section 4.8) of the Vive system were corrected. The analysis comprised a processing pipeline with several steps for re-referencing, filtering and correction of offsets. Processing steps for temporal synchronization and re-references are only relevant for this comparative study and can be neglected when using the Vive system in a production system. These steps are marked below with (ref) and (sync), respectively.

The processing runs through the following steps:

1. Shift of the origin (ref).
2. Derivation of joint angles.
3. Resampling (sync), filtering and interpolation.
4. Offset correction (ref).
5. Shift of time offset (sync).
6. Data segmentation and annotation.
7. Dealing with time interruptions (sync).
8. Systematic correction.

The whole analysis was implemented in Python version 3.7.4 and supported with the toolboxes numpy 1.19.3, scipy 1.15.0, pandas 1.2.0, matplotlib 3.2.1 and seaborn 0.9.0.

### 4.1. Shift of the Origin

In order to be independent of a global coordinate system, the origin of the joint positions of both systems was initially set to the center of the hip. All other joint positions were then oriented to the hip’s center. This step was for visualization purposes only and allowed the kinematic data of both systems to be displayed in a unified coordinate system (see Figure 3b).

### 4.2. Derivation of Joint Angles

**Figure 4:**
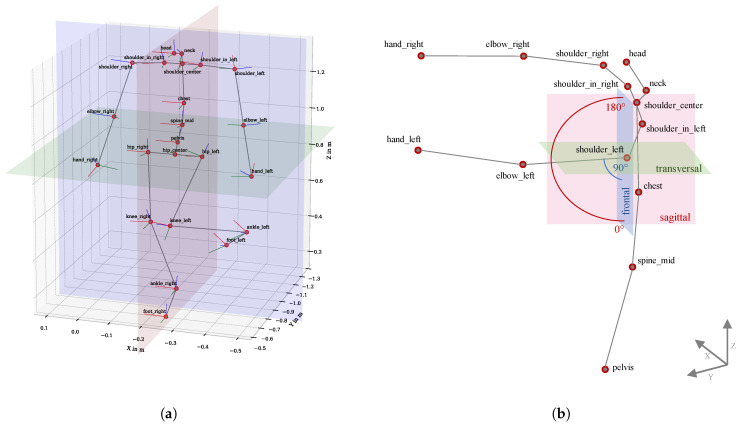
Method for calculating joint angles. (**a**) Kinematic data (positions and local coordinate systems based on quaternions) from body posture when standing on one leg while the right hand rotates. Globally plotted body planes: frontal in blue, sagittal in red and transversal in green. (**b**) An example of the calculation of the left shoulder’s elevation (*shoulder_left_elev*). The body planes are set locally to the *shoulder_left* position and orientated using *shoulder_center* and *chest*. The elevation angle is calculated with the frontal plane and moves in the sagittal plane.

According to [25]. joint angles are generally referenced to body planes. The body planes are those that intersect the body frontal, sagittal and transversal planes [26]; see Figure 4a. When interpreting movements of the whole body, it is necessary that the respective body planes tilt or rotate based on the respective body postures. To achieve this based on kinematic data, specific joints were selected to attach the planes locally. This allowed the calculation of angles between a limb and a body plane. For example, for the elevation of the left shoulder, the frontal plane is orientated by the *shoulder_center* and *chest* position, and attached locally to the *shoulder_left* position. Then the elevation angle of the *elbow_left* position with reference to the frontal plane, lying within the sagittal plane can be calculated, as shown in Figure 4b. The direction of movement was determined using the cross product of plane and joint positions. For example, a straight-forward pointing arm would have a shoulder elevation angle of $+90^{\circ }$; a straight-backward pointing arm would have a shoulder elevation angle of $-90^{\circ }$. See Table 2 for a list of the investigated joint angles with information about the chosen body planes and local orientations.

**Figure 5:**
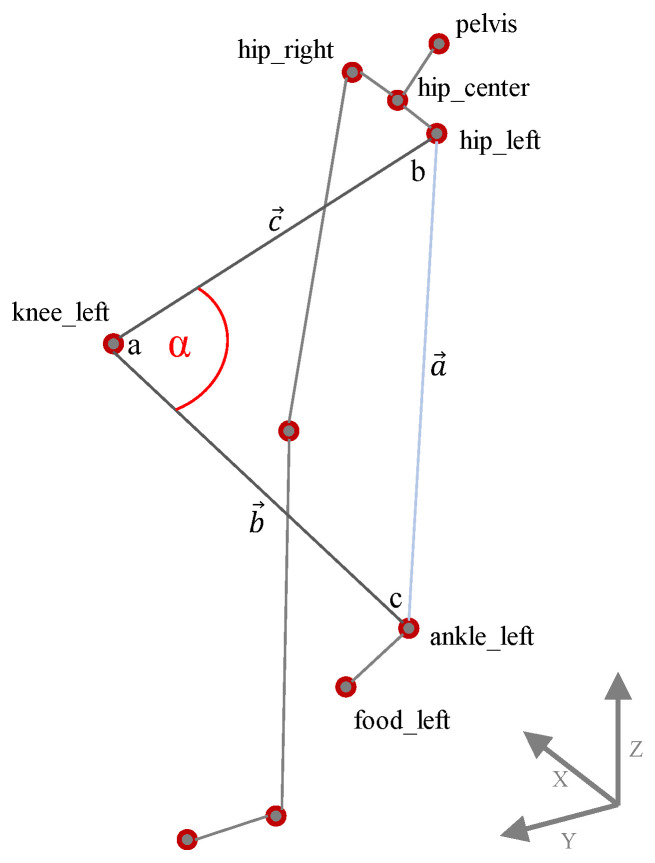
Calculation of flexion angles based on three Cartesian joint positions (x, y, z) using the example of left knee flexion.

The flexion angles *knee_(left, right)_flex and elbow_(left, right)_flex* can directly be calculated from three Cartesian coordinates without using body planes. An illustration of the calculation scheme using knee flexion as an example can be seen in Figure 5. First the vector lengths between hip and ankle, knee and ankle and hip and knee—or $\vec{a},\vec{b}$ and $\vec{c}$—need to be calculated using Equation (1). Subsequently, the angle α in position *a* and thereby the flexion in the knee joint can be determined using Equation (2) [27,28].

(1) $$v\left(P_{1},P_{2}\right)=\sqrt{\left(x_{2}-x_{1}\right)+\left(y_{2}-y_{1}\right)+\left(z_{2}-z_{1}\right)}$$

(2) $$\alpha =\arccos\left(\frac{{\vec{a}}^{2}-{\vec{b}}^{2}-{\vec{c}}^{2}}{-2\cdot \vec{b}\cdot \vec{c}}\right)$$

For the last joint in one bone connection such as that to the head, hand or foot, the quaternions (qw, qx, qy, qz) must be used to derive the angle. This is done by deriving the relative rotation based on the quaternions of the specific joint and its predecessor joint. The relative rotation between two quaternions is calculated using Equation (2). For the head, for example, the predecessor would be the neck. Quaternions of the target joint $q_{1}$ are inverted and multiplied ⊗ by those of the predecessor joint $q_{2}$, resulting in the relative rotation between the two joints, expressed as quaternion $q_{1}$ [29].

(3) $$q_{12}=q_{1}^{-1}⊗q_{2}$$

The relative quaternions can be transformed into Euler angles, so that the respective rotation or flexion can be determined [30]. In this study, the quaternion-based method was used only for the head angles. Angles of the hands (rotation and tilt) were not evaluated in this analysis. Due to the controller’s grip, it is not possible to adopt a natural hand position. The joint positions and a local, right-hand rotating coordinate system based on the quaternions are shown in Figure 4a. For each joint angle a calculation equation was implemented with the specific allocation of the body planes. The angles were implemented according to the requirements of RULA [5] and REBA [7].

### 4.3. Resampling, Filtering and Interpolation

The two systems recorded at different sample rates. For the comparison, Vive (90 Hz) was resampled at Qualisys’s sample rate (100 Hz) using a Fourier-based interpolation [31]. Isolated samples with tracking problems were interpolated. Furthermore, the signals of both systems was cleaned from high frequency noise by using a second-order low-pass filter with a cutoff frequency of 3 Hz.

### 4.4. Offset Correction

Joint angles are usually assigned to a specified RoM. An angle of zero is chosen for such a posture so that an easy interpretation of the angle is possible; e.g., elevation of the shoulder is zero in case that the arm is hanging downward, parallel to the central body’s axis. According to the neutral-zero method (NZM), all joint angles are in zero positions when a person is in a relaxed posture with arms hanging down, palms of the hands pointing to the body, shoulders wide, feet forward and an upright head position [32]. This posture is individual for each person due to different body constellations. In order to set a uniform starting point in the RoM, an offset must be identified for each joint angle. Therefore, at the beginning of each measurement, the subject was asked to remain in this neutral-zero position for at least five seconds. An offset value per angle was derived based on the average of the first two seconds of this time slot. All angles were corrected by this offset to start the movements with defined angle values. This posture was also resumed after each movement segment. Each movement ended in the neutral-zero position. This ensured that a segment sequence forced the angles of both systems to $0^{\circ }$ at the beginning of a segment sequence.

### 4.5. Shift of the Time Offset

A comparison of time series assumes time synchrony. The time series of both measurement systems were recorded simultaneously, but not time synchronously, as there was no direct communication between the systems, being that they are both commercial products. Corrective steps were performed to reconstruct time synchrony. To determine the time offset, a synchronization movement (T-posture) was performed at the beginning of the measurement.

### 4.6. Data Segmentation and Annotation

**Figure 6:**
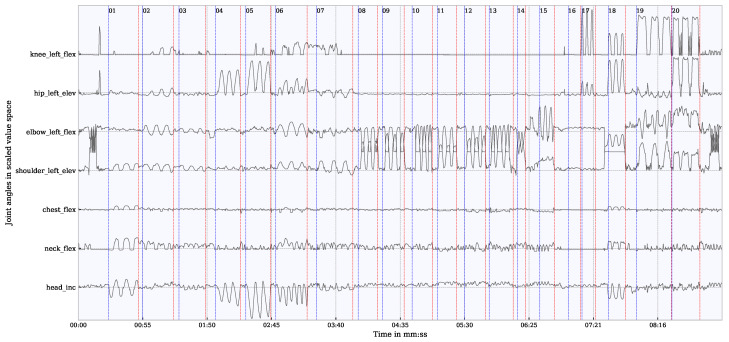
Time series of some exemplary selected joint angles. Time segments of the examined movements (01–20) are marked as vertical lines. Start of a movement in blue; end of the movement in red.

A video was recorded in parallel to the motion tracking using Qualisys and Vive. Based on the video information, the time series was divided into segments of specific movements. These segments were annotated with the specific movements and transferred to the time series of both measurement systems. The beginnings and endings of these temporal segments are shown with blue and red vertical lines in Figure 6. Each specific movement is linked to one or more joint angle in the analysis. In the comparison of the joint angles only these segments which show movements linked to the specific joint angles were considered to exclude long phases of non-movement which would have reduced the error statistic. For example, for the evaluation of the shoulder angle, only the segments in which the shoulder moved were considered. An example of a complete measurement of a participant with selected joint angles of Vive is shown in Figure 6.

### 4.7. Dealing with Time Interruptions

After the processing steps (1) to (6) as described before, the time series should be synchronized. However, time delays between the time series of Vive and Qualisys still occur for some participants. Therefore, a second synchronization process was performed, as otherwise a time shift would have a direct influence on the angle differences, although the angle itself could be accurate (but show up in another moment of time). This is due to short time interruptions in the time vector and in the time series data of the Vive system. These interruptions could be tolerable (if angular velocities are neglected) in using just one measurement system to determine tracking data, but for the comparison of the angles these shifts would influence the derived accuracy. Therefore, both signals should be synchronized as well as possible. For this reason, synchronization correction was calculated for each motion segment (i.e., for each specific movement), as explained in the following.

**Figure 7:**
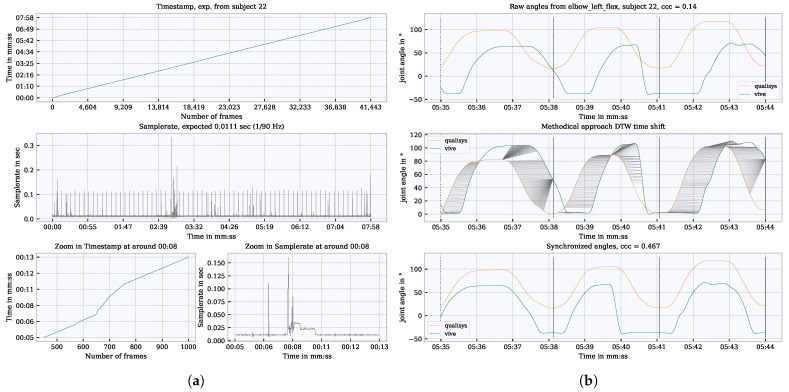
Cause of the occurring time shifts and demonstration of resynchronization. (**a**) Sample rate irregularities when recording kinematic data with Vive over an entire measurement per subject for a longer times series and zoomed in. (**b**) Representation of a time shift between Vive and Qualisys, and the synchronization approach based on DTW; black dotted vertical lines show the segment boundaries of a movement.

A closer look into the Vive time vector shows irregularities in the 90 Hz (0.011 s step width) sample rate. Examples of measurement interruptions are shown in Figure 7a. The resulting time shift between Qualisys and Vive at, e.g., the left elbow flexion angle, is shown in the top graph in Figure 7b. The segment boundaries refer to the time vector of the Qualisys with respect to the video recording. The task of the synchronization is to shift the Vive signal so that it is synchronized with Qualisys within the segments. To achieve this, two approaches were implemented which search for shifts in several iterations from rough to fine. Rough time shifts (0.5 s <shift< 2 s) can be identified by an approach based on the dynamic time warping (DTW). In general, DTW is used to identify commonalities between signals [33]. In the process, so-called pairs are formed, i.e., points between two signals that match each other. In order to measure a time offset from this, the pairs that lie on a horizontal plane in relation to each other can be used. Then an average value is calculated from the horizontal pairs, and this value is used for shifting. Due to the averaging in the method, the measured offset is a rough approximation, which is still not accurate enough. This approach is shown in the middle graph of Figure 7b.

In order to implement the synchronization even more precisely, a second fine (shift< 0.5 s) approach has been applied. This was based on the fast Fourier transform (FFT) and searched for a time offset at the phase shift between two signals. Using both methods, it was possible to resynchronize the signal after an interruption caused by a measurement dropout. The resynchronized signal can be seen in the bottom graph in Figure 7b.

### 4.8. Systematic Correction

**Figure 8:**
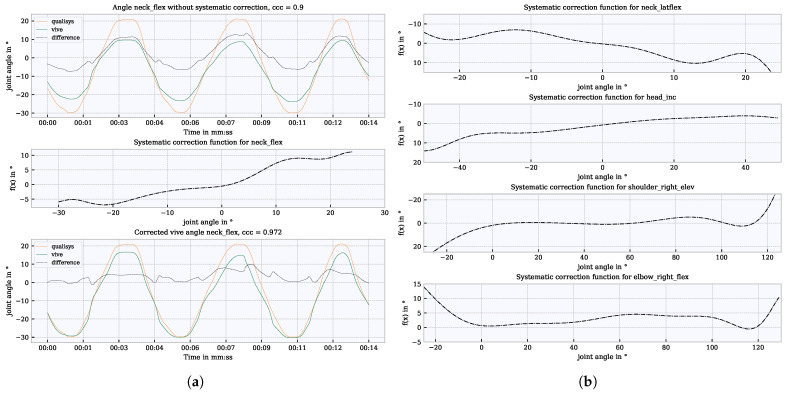
Systematic correction by polynomial regression function. (**a**) Systematic differences using the example of neck flexion (upper graph); correction function (middle graph); corrected joint angle (lower graph); (**b**) further correction functions using the 3 tracker configuration as an example.

Both systems used IK for the calculation of the kinematic data. The sensor technology and the calculation basis for the IK differed. Based on this, for some joint angles, systematic deviations (i.e., an overestimation or underestimation) between Vive and Qualisys were found (see Figure 8a, upper graph). Therefore, a polynomial regression function was used to correct systematic deviations in regard to the angle values. This correction function was fitted with the data of all subjects and applied for each individual subject according to the following processing steps:

1. Vive: concatenate time series for each subject.
2. Qualisys: concatenate time series for each subject.
3. Select only relevant temporal segments for each joint angle.
4. Calculate differences between the systems.
5. Fit the regression function with the differences and corresponding Vive angle for each joint angle.

First, the time series of the respective joint angles were concatenated with the data from each subject using only the time segments in which the joint was moved. This step was performed for both systems. Subsequently, the differences (offsets) between the systems were determined. With the difference values and the corresponding angles of Vive, a polynomial regression function was then fitted. The determination of the degree of polynomial regression was studied iteratively, and the degree with the best accuracy (12th degree) was used. This function was then used to correct the joint angles for each individual subject. The systematic correction function and the corrected joint angle for the neck flexion angle are shown in the middle and lower diagrams of Figure 8a. The correction functions for *neck_latflex*, *head_inc*, *shoulder_right_elev* and *elbow_right_flex* are shown in Figure 8b to give examples of differently shaped correction functions.

## 5. An Approach to Compare Systems based on Joint Angles

The data analysis focused on the comparison of the sample-wise calculated joint angles of Vive (with FinalIK) and Qualisys. For the evaluation, only the temporal segments in which the respective joint was moved were selected to avoid non-movement phases which would improve the overall error statistic. The segments, selected that way, were concatenated across all subjects. Three consecutive segments of a random subject’s preprocessed time series (Qualisys and Vive) and the differences in neck flexion are shown in the lower graph of Figure 8a.

### 5.1. Violin Plots

**Figure 9:**
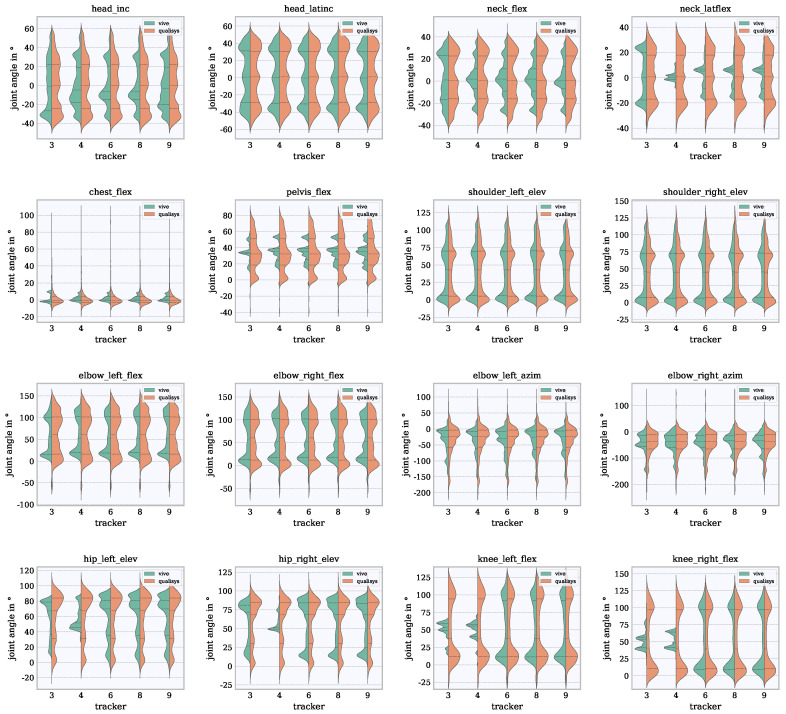
Violin representation of the examined joint angles of the Vive tracker configurations 3, 4, 6, 8 and 9 (left violin, green) in comparison to Qualisys (right violin, orange). The quartiles (25%, 50% and 75%) are shown as dotted lines.

**Figure 10:**
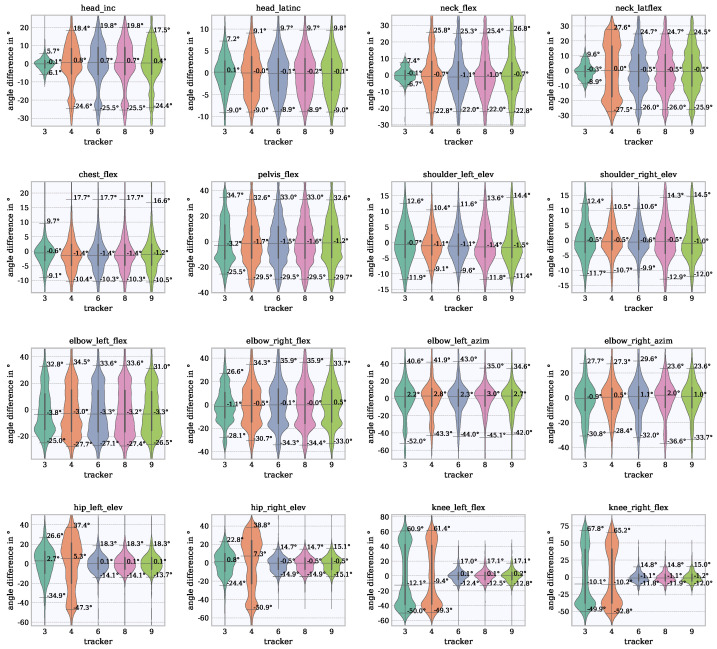
Comparison of the frequency of occurring angle differences and a comparison of the tracker configurations.

The sample-wise joint angle differences and their distribution are considered to assess the accuracy of the Vive combined with Final IK. The differences are shown in violin plots in Figure 9 and Figure 10. In Figure 9, the incidences of joint angles are shown with respect to the RoM. It can be seen whether certain angles in the RoM are frequently or rarely reached with both systems. Ideally, the shape of the Vive violin on the left is the same as the shape of the Qualisys violin on the right. This plot includes quartiles (25%, 50% and 75%) which provide indications of the frequencies of joint angles that occurred. In Figure 10, the incidences of joint angle differences are shown. This plot provides information about the value ranges in which the differences accumulated and which differences occurred more rarely. For a better elaboration of the range of values in which the differences varied and for the elimination of outliers, percentiles between 5% and 95% were calculated. The accuracy was described by the inter-percentile-range (IPR) of the 5th and 95th percentiles. The orientation of the median value indicates whether an angle is rather overestimated or underestimated. A median value at $0^{\circ }$ would mean that the difference is equally overestimated and underestimated or suggests that the signals match very closely.

### 5.2. Bland–Altman Plots

A common analysis method to compare two signals or measuring methods is the Bland–Altman plot [34]. In this study, the Bland–Altman plot was modified. As we assumed that the Qualisys data were much more accurate than the Vive data, and the differences were relatively large, we decided to plot only the Qualisys reference data on the x-axis instead of the average of both systems. This allows one to see in which angular range the difference occurs. On the y-axis the difference between both systems is shown. The limits of agreement (LoA) are displayed as Mean±1.96×standard_deviation. The Bland–Altman plots are represented with 2500 randomized (normally distributed with unique index values) difference points from the time series.

### 5.3. Concordance Correlation Coefficient

Using the concordance correlation coefficient (CCC) according to [35], the correlation between two systems can be calculated. The CCC can be seen as an extension of the Pearson correlation [36], since in addition to the correlation, a location and time shift is also taken into account. The fundamental calculation of the CCC is shown in Equation (4).

(4) $$ccc=\frac{2\sigma_{qv}}{{\left(\mu_{q}-\mu_{v}\right)}^{2}+\sigma_{q}^{2}+\sigma_{v}^{2}}$$

where $\sigma_{qv}$ is the covariance, $\mu_{q},\mu_{v}$ are the mean values and $\mu_{q},\mu_{v}$ are the standard deviations from *q* Qualisys and *v* Vive. According to [37], correlations <0.90 are evaluated as poor, >0.90 to <0.95 as moderate, >0.95 to <0.99 as substantial and >0.99 as almost perfect. An example of how the CCC behaves with a scale shift between two signals can be explained with the help of Figure 8a. In the upper graph, the CCC is 0.9 for a scale-shifted signal (Vive, Qualisys). The lower graph shows that the CCC increases to 0.978 when the shift between the signals is corrected.

### 5.4. Plausibility Check Based on WIDAAN

The results based on WIDAAN were used to cross-check the processing and the results as a plausibility check. It is expected that despite differences in processing to derive joint angles, equal trends of joint angle differences will be seen in the results.

WIDAAN was established for the evaluation of workplaces based on ergonomic criteria [8]. In order to produce comparable results independent of individual body sizes, the data are mapped to a body model, the so-called “the Dortmunder” [38]. This methodology differs from the analysis in this paper in the sense that the individual’s body model, i.e., the individual antrophometric dimensions of the respective subject, is used for the angle calculation. Another difference is that in WIDAAN the joint angles are restricted to plausible angle ranges for the respective joint angles. The method in this paper always considers the actual measured angle based on the kinematic data according to Section 4.2. Furthermore, a correction function for the adjustment of systematic offsets was applied in this study (Section 4.8). The main differences between the approach in this paper and WIDAAN are listed in Table 3. Table 4 shows a list of angles which, despite the processing differences, should indicate a similar trend of calculated differences obtained with WIDAAN and the approach in this paper.

## 6. Results of the System Comparison

In this section, the results of the comparison of sixteen joint angles between Vive and Qualisys are presented. Five different tracker configurations (3, 4, 6, 8 and 9) of Vive were compared with Qualisys in each case. The Qualisys system was used as ground truth. A general overview of the analyzed joint angles with 25%, 50% and 75% quartiles and the mean value, standard deviation minimum and maximum, is shown in Table A1 in Appendix A. In the following, the results based on the violin plots, Bland–Altman plots and the CCC are presented.

### 6.1. Distribution of Joint Angles in the RoM Based on Violin Plots

The violin plot of the analyzed angles for Qualisys and the five Vive tracker configurations can be seen in Figure 9. The tracker configurations 3 and 4 differ from tracker configurations 6, 8 and 9. From 6 onward (configurations 6, 8 and 9), no differences are noticeable. There are no essential differences between left and right limbs; therefore, both (left and right) joint angles are combined in the following.

For *head_latinc*, *chest_flex*, *pelvis_flex*, *shoulder_elev*, *elbow_flex* and *elbow_azim* there are no differences between any tracker configurations. Considering the head and neck angles, a higher agreement with Qualisys can be seen in tracker configuration 3 compared to the other configurations. Furthermore, very similar shapes are shown for *head_inc*, *shoulder_elev* and for *knee_flex* from configuration 6 onward compared to Qualisys. *elbow_flex* and *elbow_azim* show similar shapes to the results in Qualisys but a lower RoM for all tracker configurations. In *elbow_flex* the shape of Vive shows a peak at about $10^{\circ }$, while Qualisys sets this peak at about $0^{\circ }$. *hip_elev* shows a similar shape compared to the shape made by Qualisys from configuration 6, but with a lower RoM. *knee_flex* shows a good agreement with Qualisys for tracker configurations 6, 8 and 9. *pelvis_flex* shows a lower RoM and a different shape than Qualisys.

### 6.2. Joint Angle Differences Based on Violin Plots

A violin plot of the differences between Vive and Qualisys compared across the tracker configurations with 5th and 95th percentiles, and the median values, can be seen in Figure 10.

From tracker configurations 6, 8 and 9, there are no differences. In general, the joint angle differences can be divided into good ($0^{\circ }$ to $\pm 10^{\circ }$), medium ($\pm 10^{\circ }$ to $\pm 15^{\circ }$) and poor (>$\pm 15^{\circ }$) based on the IPR range.

- Good: three tracker configurations of head_inc, head_latinc, neck_flex, neck_latflex and chest_flex.
- Medium: shoulder_elev with all tracker configurations.
- Poor: pelvis_flex, elbow_flex, elbow_azim, hip_(left, right)_elev and knee_flex with all tracker configurations.

Generally, the IPR ranges from $\pm 6^{\circ }$ to $\pm 42^{\circ }$. Considering the angles of the head, neck, chest, shoulders, hips and knees (configurations 6 and above), the IPR were between $\pm 6^{\circ }$ and $\pm 18^{\circ }$. The IPR of the elbows and pelvis were between $\pm 30^{\circ }$ and $\pm 42^{\circ }$.

### 6.3. Correlation Based on the CCC

**Figure 11:**
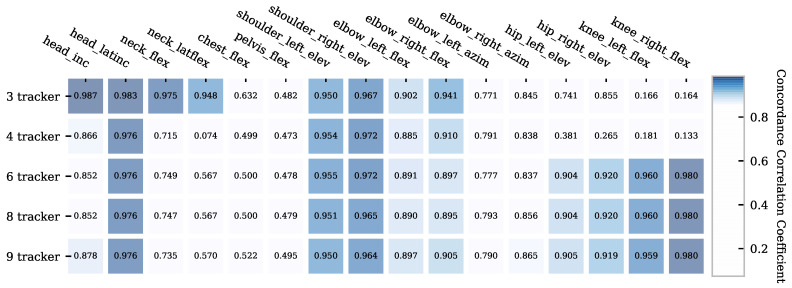
Concordance correlation coefficient for all studied joint angles and tracker configurations.

The CCC comparing the joint angles and tracker configurations with color highlighted correlations greater than 0.85 are shown in Figure 11. According to McBride [37], *head_inc*, *head_latinc* and *neck_flex* achieve a substantial correlation with three trackers and a poor one with four trackers or more. The *shoulder_elev* reached a substantial correlation for all tracker configurations. The *elbow_flex* angles achieved a moderate to poor correlation for all tracker configurations. Furthermore, a moderate to substantial correlation can be seen in the hip and knee angles for tracker configurations 6 and above. *Chest_flex*, *pelvis_flex* and *elbow_azim* show poor correlations in all tracker configurations. *hip_elev* reached a moderate correlation for tracker configurations 6 and above. For *knee_flex*, a substantial correlation can be seen for tracker configurations 6 and above.

### 6.4. Assessment of Accuracy Using Bland–Altman Plots

Bland–Altman plots for the joint angles *neck_flex, shoulder_left_elev, elbow_left_flex* and *knee_left_flex* across the studied tracker configurations are shown in Figure 12, Figure 13, Figure 14, Figure 15 and Figure 16. The Bland–Altman plots for the further twelve studied joint angles are shown in Figure A1, Figure A2, Figure A3, Figure A4 and Figure A5 in Appendix A.

**Figure 12:**
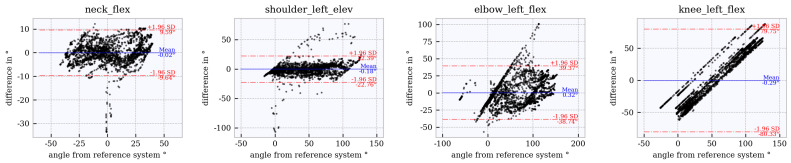
Bland–Altman plot for **tracker configuration 3**; x does not show the average of both systems but the joint angles of the reference system, Qualisys.

**Figure 13:**
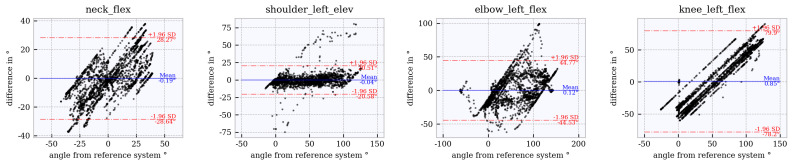
Bland–Altman plot for **tracker configuration 4**; x does not show the average of both systems but the joint angles of the reference system, Qualisys.

**Figure 14:**
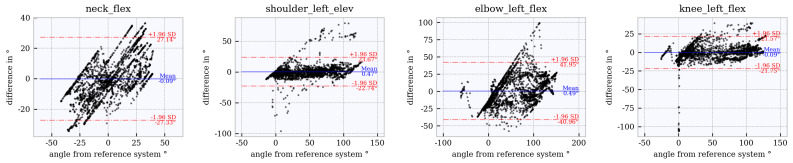
Bland–Altman plot for **tracker configuration 6**; x does not show the average of both systems but the joint angles of the reference system, Qualisys.

**Figure 15:**
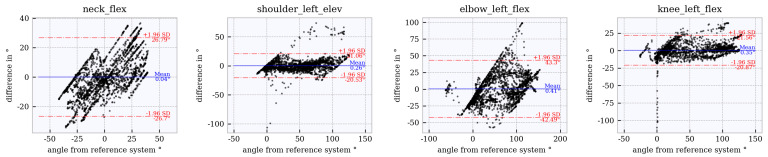
Bland–Altman plot for **tracker configuration 8**; x does not show the average of both systems but the joint angles of the reference system, Qualisys.

**Figure 16:**
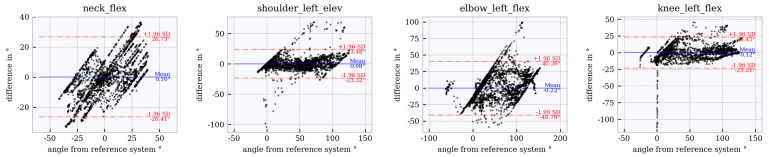
Bland–Altman plot for **tracker configuration 9**; x does not show the average of both systems but the joint angles of the reference system, Qualisys.

The mean of all angles and tracker configurations was approximately $0^{\circ }$. The LoA (red dotted lines) for *neck_flex* in tracker configuration 3 shows a value of approximately $\pm 10^{\circ }$ (Figure 12). This range increases to approximately $\pm 27^{\circ }$ (Figure 13, Figure 14, Figure 15 and Figure 16) for the other tracker configurations, showing a trend to a linear deviation. For *neck_flex* (three trackers) and for *knee_left_flex* (from six trackers), differences at $0^{\circ }$ can be seen in isolated cases. At *shoulder_left_elev* and *elbow_left* the LoA does not vary between the tracker configurations (approximately $\pm 23^{\circ }$ and $\pm 42^{\circ }$, respectively). In the *knee_left_flex* angle, tracker configurations 3 and 4 show linear deviation over the entire measuring range of the reference system. For tracker configurations 6, 8 and 9, the LoA remains constant at $\pm 17^{\circ }$. The occurrence of linear deviations can also be observed when considering *pelvis_left* in all five configurations and at the *hip_elev* and *knee_flex* in configurations 3 and 4 (see Appendix A).

### 6.5. Comparison to WIDAAN

**Figure 17:**
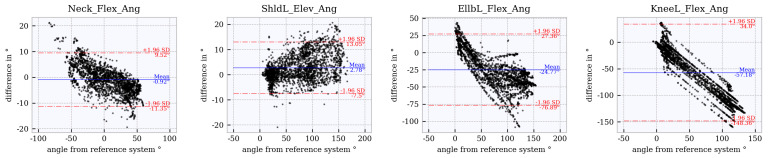
Bland–Altman plot for **tracker configuration 3 from WIDAAN**; x does not show the average of both systems but the joint angles of the reference system, Qualisys.

**Figure 18:**
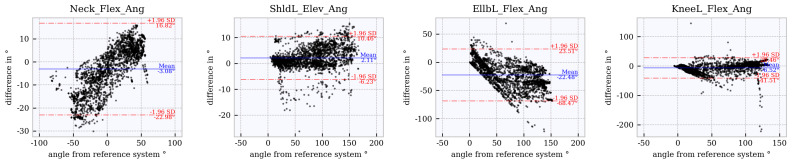
Bland–Altman plot for **tracker configuration 9 from WIDAAN**; x does not show the average of both systems but the joint angles of the reference system, Qualisys.

**Figure A6:**
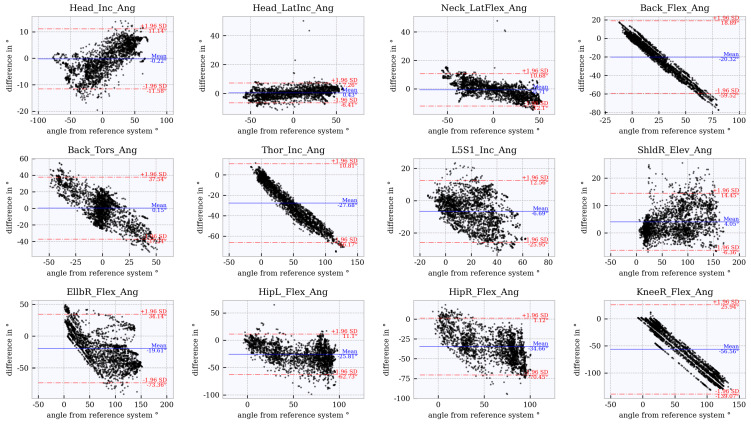
Bland–Altman plot for **tracker configuration 3 from WIDAAN**; x does not show the average of both systems but the joint angles of the reference system, Qualisys.

**Figure A7:**
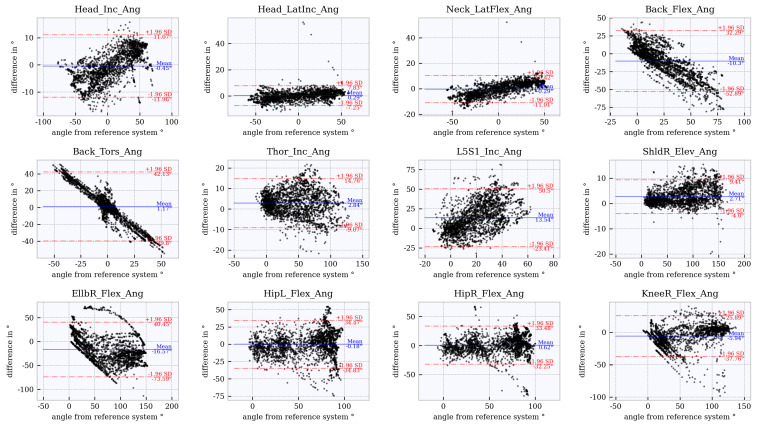
Bland–Altman plot for **tracker configuration 9 from WIDAAN**; x does not show the average of both systems but the joint angles of the reference system, Qualisys.

The results calculated using the approach in this paper are compared with those calculated by WIDAAN: Bland–Altman plots based on joint angle differences calculated with WIDAAN for tracker configurations 3 (Figure 17) and 9 (Figure 18) are compared with the same joint angles as in Section 6.4. Bland–Altman plots of further joint angles calculated by WIDAAN can be found in Figure A6 and Figure A7 in Appendix A. A direct comparison between the results from this paper and WIDAAN, based on mean and LoA, can be seen in Table 5 and Table 6. Differences between the approach in this paper and WIDAAN based on the Bland–Altman plots for the neck flexion, left shoulder elevation, left elbow flexion and knee flexion is described below.

Neck flexion (configurations 3 and 9): The mean and LoA show no major differences between the approaches when considering the tables. This consideration is valid for both tracker configurations. Comparing the tracker configurations 3 and 9 in the WIDAAN Bland–Altman plots, a downward linear deviation occurs with configuration 3, whereas configuration 9 shows an upward linear deviation. Left shoulder elevation (tracker configurations 3 and 9): It is noticeable that the range of values in the LoA of WIDAAN is smaller by about $10^{\circ }$. The mean value shows negligible differences. This trend is similar for both configurations. Left elbow flexion (tracker configurations 3 and 9): The LoA shows a large range of values for both approaches. Regarding the mean, it can be seen that the approach in this paper reached around $0^{\circ }$, and for WIDAAN the mean value was smaller than $-20^{\circ }$. Knee flexion (configuration 3): Linear deviations, because the legs are not captured with tracker configuration 3. Considerable differences in the mean between the approaches. Knee flexion (configuration 9): In tracker configuration 9, it can be seen that the mean was slightly decreased by WIDAAN. Looking at the lower LoA, it can be seen that WIDAAN set a noticeably lower value.

In summary, the mean value for the approach in this paper was around $0^{\circ }$, whereas for WIDAAN, high deviations in the mean value can be seen for elbow and knee flexion. Both approaches show similar trends for angles that also showed good accuracy (e.g., neck flexion). For angles with generally poor accuracy (e.g., left elbow flexion), higher differences between the approaches can be seen.

## 7. Discussion

The discussion is structured as follows. In Section 7.1 tracking and data problems are discussed. In Section 7.2 technical and physical differences between the systems are addressed. Differences between the tracker configurations are discussed in Section 7.3. The accuracies per joint angle are in focus in Section 7.4.

### 7.1. Tracking and Data Problems

**Figure 19:**
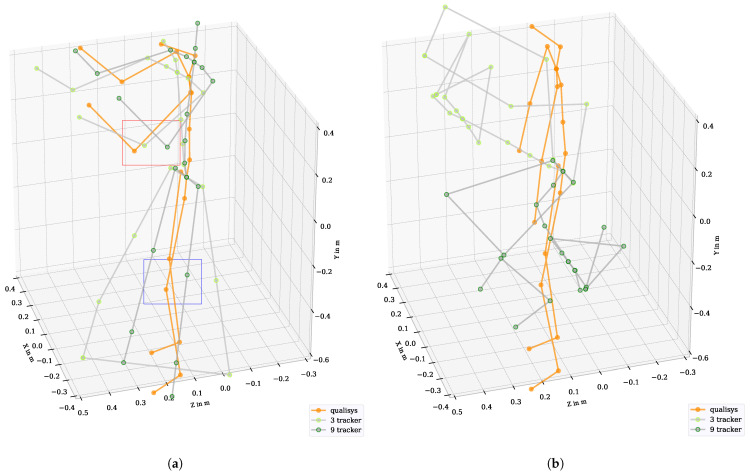
Tracking problems identified by comparing the kinematic data between Qualisys and Vive in tracker configurations 3 and 9. (**a**) Kinematic differences with a focus on the elbow between tracker and Qualisys (red box). Error based on a rigid knee in trackers (blue box). (**b**) Illustration of an implausible representation of kinematic data by tracker data.

Three different types of tracking errors have been found. In one type, isolated limbs appear to remain rigid even though movement is taking place, which is especially evident for short movements. This error can be seen in the blue box in Figure 19a, where the knees show flexion for Qualisys but the knees with the Vive tracker configuration 9 remain straight. This error leads to linear differences that can be seen in the Bland–Altman plots in, e.g., Figure 12 at *knee_left_flex*.

Another error is shown by individual joint positions or all joints jumping to implausible values (see Figure 19b). This is represented by differences at $0^{\circ }$ in the Bland–Altman plots, e.g., at *neck_flex* at $0^{\circ }$ (see Figure 12). This situation could only be observed in one subject when the subject was standing quietly in the neutral position. Two subjects were removed from the study because they showed a high number of short tracker jumps (e.g., the arm jumps through the body to the back and forward). These jumps could be caused by either a calculation error in Final IK or tracking loss, which was already mentioned in [9]. Tracking loss can happen due to the occlusion of trackers by limbs during the movement. However, we already used four base stations in our set up to reduce the probability of occlusion. Consequently, we suggest the implementation of plausibility checks before conducting the analysis to reduce the influence of tracking errors. Therefore, implausible data were removed or corrected before further analysis.

**Figure 20:**
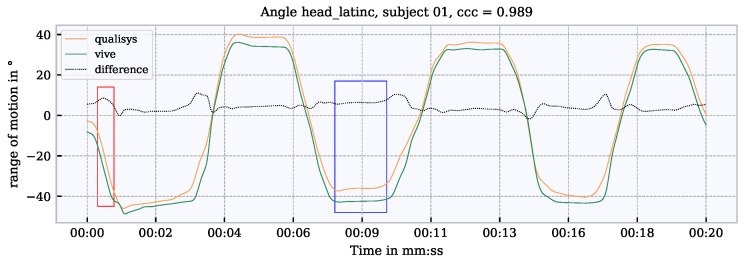
Differences caused by time shifts (red box). Differences which are relevant for the accuracy (blue box).

Another possible source of error could be the setup with the two measurement systems themselves. Both systems, Qualisys and Vive, use infrared pulses for distance measurement. Although care was taken to ensure that the systems were not in direct line of sight of each other, inference cannot be ruled out. A similar observation was also seen in [18]. Therefore, we think that fewer tracking errors will occur if only the Vive system is used and suggest other tracking methods for further studies (e.g., IMU based systems/XSENS). In addition to the tracking errors, we found a loss of data points due to measurement interruptions, which are described in Section 4.7. It can be assumed that, as in [9], loss of tracking can lead to short interruptions. As this missing data could lead to asynchrony between Vive and Qualisys, we used resampling, interpolation and resynchronization based on the DTW and FFT methods to correct these time shifts. Even after these corrective steps, small time shifts appeared which led to differences in the angle data for fast joint movements (i.e., high angle slopes). Differences caused by overestimation and underestimation and by time shifts are shown in Figure 20.

Due to data loss, datasets from four subjects could not be saved at all or without errors (missing tracker configurations). It should be noted that the system usually generates kinematic data for only one tracker configuration. The special case in this study allowed data to be saved for all configurations simultaneously. Nevertheless, the larger amount of data led to unresolved errors during the saving process. However, saving the data was triggered after the measurement and therefore should not have been the cause of problems that occurred during the measurements themselves.

### 7.2. Systematic Differences

In our analysis, we found systematic differences between the Qualisys and Vive angles, shown by underestimation and overestimation of the joint angles of the Vive data. In both systems, an IK is used to calculate the kinematic data from motion capture data. However, the kinematic data differed technically in the resulting body models. They showed differences in the positioning, e.g., of neck and chest joints (see Figure 3b). It was first assumed that the offset correction described in Section 4.4 would resolve these differences. However, even after the offset correction, consistent underestimations and overestimations of the angles were found in the entire RoM (see Figure 8a). We also found these systematic differences (i.e., underestimation and overestimation) in our WIDAAN analysis, where the same body model was used to calculate the Qualisys and Vive angles. Consequently, we assumed that these dynamical differences were caused by the IK calculation of Final IK. We therefore used the systematic correction function described in Section 4.8 to correct these differences. For the head and neck angles, each underestimation was compensated almost linearly. In the maximum and minimum ranges of the RoM, each function became flatter. For shoulder and hip elevation, Vive did not reach the full minima and maxima of RoM. The correction functions show increases in the correction factor at the border ranges. For angles with poor accuracy, such as the elbow, chest and pelvis, systematic correction can only limit major outliers. This correction achieved an improvement in the order of about $\pm 5^{\circ }$ for joint angles such as head and neck flexion. For angles such as the elbow with inaccuracies >$\pm 25^{\circ }$, only very high outliers could be limited. Nevertheless, it must be noted that the function was trained with motion data from this analysis. Further testing on random movements needs to be done. Systematic differences were also found in [13] when using several base stations on a position basis. Since we also worked with four base stations, it cannot be ignored that this error also occurred in this analysis when calculating the joint angles.

Another possible explanation of some systematic differences (e.g., elbow) is the slippage of a tracker. In Qualisys, the IK is performed with positions from marker points fixed on the skin. For Vive, IK is calculated using positional data from trackers attached to limbs with straps. It is assumed that the trackers can slip on the limbs in certain movements. For an evaluation of the effects of sensor slippage, it should be further investigated to what extent and during which movements a physical slippage of the Vive tracker occurs and how this also affects the calculation of the kinematic data with Final IK.

### 7.3. Differences between the Tracker Configurations

Joint angles for five tracker configurations were analyzed and compared. In tracker configuration 3 only the head and arms were captured. In configuration 4 the torso was tracked, and from configurations 6, 8 and 9 the legs are represented. Configurations 8 and 9 were assumed to improve the accuracy of the upper body. One might assume that more trackers would improve the accuracy. However, we only found differences in tracker configurations for the head and neck angles, which showed the best results in tracker configuration 3, and for the hip and knee angles. Our results have shown that the hip and knee angles can only be considered for configurations 6, 8 and 9 (i.e., with one tracker on each foot). The more trackers used, the more angle solutions that have to be found by the IK. Therefore, we assume that there is no advantage in using the 8 or 9 trackers in comparison to the 6-tracker configuration. However, if the focus is on the head and neck angles, the 3-tracker configuration works best. Additionally, it should be considered that the chance of tracker slippage raises with every extra tracker. Such a slippage not only influences the joints of the specific segment but also all joints based on the IK.

However, in our analysis we only examined the time segments in which an intentional movement of the joint was performed. Due to the IK calculation, joints move, although they are actually at rest if only few trackers are used. Using less tracker can therefore lead to incorrect results in ergonomic risk assessments.

### 7.4. Accuracy per Joint

**Figure 21:**
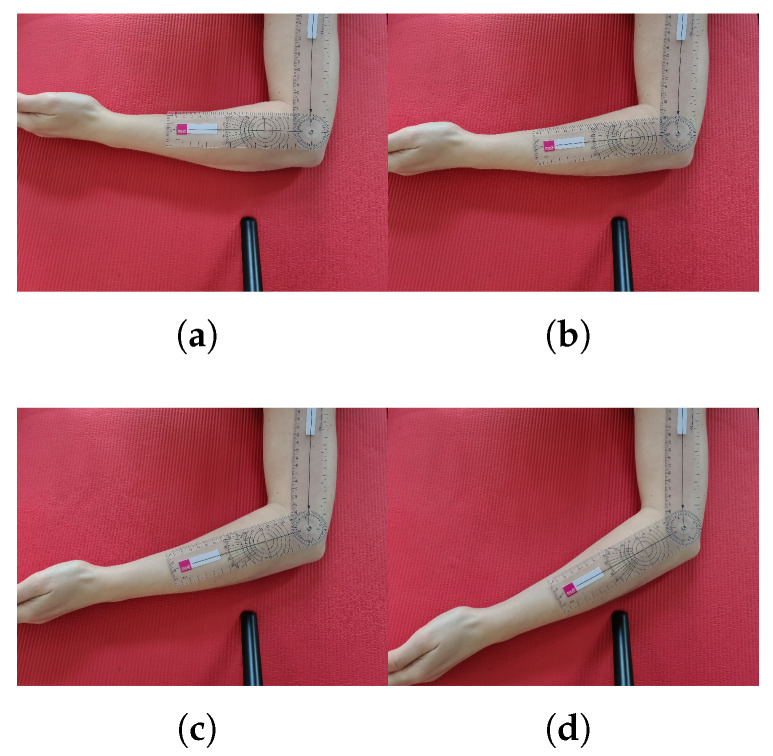
Elbow flexion from $90^{\circ }$ to $120^{\circ }$ in $10^{\circ }$ increments measured with a goniometer. (**a**) $90^{\circ }$; (**b**) $100^{\circ }$; (**c**) $110^{\circ }$; (**d**) $120^{\circ }$.

In this section, the accuracies per joint in relation to requirements for ergonomic risk assessment (RULA and REBA), based on joint angle differences in the 5–95% IPR are discussed. For a clearer image of the presented values, a visual impression of elbow flexion angles from $90^{\circ }$ to $120^{\circ }$ in $10^{\circ }$ increments, measured with a goniometer [39], is shown in Figure 21. A visualization of elbow flexion based on kinematic data comparing Qualisys and tracker configurations 3 and 9 is shown in the red box in Figure 19a. It is recommended to keep this figures in mind in order to have a real reference to the joint angle differences and causes mentioned in this paper.

Head and neck ($\pm 9^{\circ }$, three trackers): Good results are shown for the head and neck angles in the 3-tracker configuration regarding the IPR, joint angle differences and Bland–Altman plots. From the 4-tracker configuration onward the accuracy decreases. The observation in Figure 9 on the basis of the frequently achieved joint angles shows a reduction in the RoM, especially in neck lateral flexion. This is also shown in the Bland–Altman plot and led to underestimations of the head and neck angles. We assume that this underestimation is a result of the Final IK angle calculation. A higher number of trackers makes it harder for the IK to find the best fitting angle values for all angles without causing implausible data. An ergonomic assessment based on RULA [5] would provide for a threshold-based differentiation of $0^{\circ }$ to $10^{\circ }$, >$10^{\circ }$ to $20^{\circ }$ and >$20^{\circ }$ thresholds for neck flexion. Even in the 3-tracker configuration, which showed the best accuracy of the five configurations, it could only reach an IPR of <$\pm 9^{\circ }$. Therefore, we assume that the accuracy is only sufficient to distinguish between critical (>$20^{\circ }$) and non-critical. We have to further keep in mind that the head and neck angles were underestimated in the 4-tracker configuration onward, which also led to an underestimation of the ergonomic assessment.

Chest ($\pm 10^{\circ }$, three trackers): Based on our classification the chest flexion showed good accuracy. However, it must be taken into account that the RoM was between $-20^{\circ }$ and $20^{\circ }$, and based on this we should classify the chest angle as medium/poor for all tracker configurations. According to RULA, an ergonomics evaluation would be performed with a scoring in $0^{\circ }$ to $10^{\circ }$, >$10^{\circ }$ to $20^{\circ }$ and >$20^{\circ }$ to extension. It can be said that these ranges can only be assessed very roughly. High inaccuracies can also be seen in the Bland–Altman plots, in the CCC and in the back torsion, which is the corresponding angle calculated by WIDAAN on the basis of the Bland–Altman plots in Figure A6 and Figure A7 in Appendix A (LoA about $\pm 35^{\circ }$).

Pelvis ($\pm 35^{\circ }$, all trackers): Pelvis flexion shows poor accuracy for all tracker configurations across all analyzed outcome parameters. The observation in Figure 9 on the basis of the frequently achieved joint angles shows a reduction in the RoM which can also be seen as underestimations in the Bland–Altman plots. The corresponding angle (L5S1 inclination, Figure A7) calculated by WIDAAN shows large differences as well (LoA about $-12^{\circ }$ to $48^{\circ }$) for tracker configurations 3 and 9. In the REBA [7] ergonomic approach, a threshold-based evaluation with limits $0^{\circ }$ to $20^{\circ }$ and >$20^{\circ }$ to $60^{\circ }$ would be required. As this range was exceeded, the accuracy is rated as insufficient for an ergonomic evaluation.

Shoulders ($\pm 11^{\circ }$, four trackers): The accuracy of the shoulder is rated as medium. Shoulder elevation was furthermore rated with a substantial correlation between Qualisys and Vive for all tracker configurations. However, the Bland–Altman plots showed only a medium accuracy with an LoA of about $22^{\circ }$. In the ergonomics evaluation according to RULA, an angle >$45^{\circ }$ was assigned a higher score. This distinction could be achieved in the shoulder joint for all tracker configurations. However, we have to keep in mind that there are many outliers which can influence the results of the ergonomic assessment.

Elbows ($\pm 42^{\circ }$, all trackers): Elbow flexion and azimuth were rated as poor regarding the IPR for all tracker configurations. The observation in Figure 9 on the basis of the frequently achieved joint angles shows a reduction in the RoM which can also be seen as underestimations in the Bland–Altman plots. Even though the CCC for elbow flexion was rated as moderate, the accuracy of the elbows (flexion and azimuth) can generally be rated as insufficient. According to RULA and REBA, however, only a rough distinction of $0^{\circ }$ to $60^{\circ }$ or >$60^{\circ }$ to $100^{\circ }$ is required. Nevertheless, the high deviations are too inaccurate even for that.

Hip and knees ($\pm 18^{\circ }$, 6, 8 or 9 trackers): As the legs were only captured when using the tracker configurations 6, 8 and 9, the tracker configurations 3 and 4 are not sufficient for ergonomic risk assessments, and we will therefore only discuss the results from tracker configuration 6 onward. The IPR shows poor values for hip and knee angles, although the CCC shows a moderate to substantial correlation. The Bland–Altman plots show an LoA of about $\pm 20^{\circ }$. For an ergonomics assessment according to REBA, a differentiation of knee flexion of $0^{\circ }$ to $30^{\circ }$, >$30^{\circ }$ to $60^{\circ }$ and >$60^{\circ }$ would be required in order to assign a score. It can be assumed that this differentiation can only be performed very roughly.

We assume that the HMD can be tracked under the best visual conditions (no occlusion). This observation is also reflected in the accuracy of the angles for the head and neck, which were measured directly with the HMD. The observation that a higher accuracy (on positions basis) can be achieved with the HMD than with the controller and tracker was also observed in [18]. This could be due to the higher number of position sensors implemented in the HMD compared to the controller and tracker.

## 8. Conclusions

In this work, the Vive consumer motion tracker system in combination with Final IK was compared to the high precision marker-based Qualisys system on the basis of joint angles. The Vive kinematic data were calculated in five different tracker configurations (3, 4, 6, 8 and 9) and compared with the Qualisys data in each case.

We investigated how accurately joint angles can be calculated with Vive and Final IK compared to joint angles calculated with Qualisys. In total, joint angle deviations between $\pm 6^{\circ }$ and $\pm 42^{\circ }$ were identified. The second research question investigated the influence of the number of trackers on the accuracy. It can be concluded that a higher number of trackers does not lead to more accuracy. For head and neck angles, the 3-tracker configuration showed the best results. Knee and hip angles could only be calculated from the 6-tracker configuration onward. At all joint angles no differences were found between tracker configurations 6, 8 and 9. The third question investigated whether the joint angle accuracies are sufficient to perform an ergonomic analysis according to, e.g., RULA or REBA. We conclude that the joint angles calculated by kinematic data from HTC Vive and Final IK are not sufficient for ergonomic risk assessments. Causes are inaccurate joint angles, data loss and tracker position fluctuations. The highly inaccurate angles of the elbow flexion ($\pm 42^{\circ }$) and pelvis flexion ($\pm 35^{\circ }$) especially do not provide enough support for an ergonomic evaluation. The inaccuracies of these angles exceeded the ranges defined by the thresholds in the evaluation according to, e.g., RULA. Nevertheless, the system offers an attractive and cost-effective method for capturing motion data. The potential of the system was also demonstrated by the better joint angle accuracy for the head and neck.

In future work, it should be investigated whether recalibration, which was also addressed in [9], during motion capturing can improve the accuracy. Furthermore, research is being conducted into accuracy improvement by, e.g., systematic correction. Therefore, the systematic error function can be fitted with movements from this study to prove whether the same accuracies can be achieved with completely unknown or random motions. Furthermore, it should be investigated whether neglecting IK and directly deriving the joint angles from absolute values of the trackers could lead to an improvement in joint angle accuracy. It should also be investigated whether alternatives to Final IK exist and whether adaptations can achieve improvements in the kinematic data or joint angle accuracies.

## Figures and Tables

**Table 1:** Description of the movement segments and associated joint angles.

No.	Description of Movement	Used for Angle
01	head inclination	head_inc, neck_flex
02	head lateral inclination	head_latinc, neck_latflex
03	neck torsion with fixed points approx. $90^{\circ }$ at each side	
04	bending the torso forward (straight back)	chest_flex, pelvis_flex
05	bending the torso forward (curved back)	chest_flex, pelvis_flex
06	back lateral inclination	
07	back torsion with fixed points, approx. $90^{\circ }$	
08–13	lifting both arms lateral, frontal	shoulder_(left, right)_elev
14	T-pose with upper arm rotation (drawing circles with hands)	
15	elbow flexion (curls)	elbow_left, right)_flex, elbow_(left, right)_azim
16	knee flexion: standing on one leg (right), other lower leg in the air, bent backwards at a $90^{\circ }$ angle	knee_left_flex
17	knee flexion: standing on one leg (left), other lower leg in the air, bent backwards at a $90^{\circ }$ angle	knee_right_flex
18	squats with outstretched arms, back straight	
19	lunge, left leg front, knee flexion $90^{\circ }$, elbow flexion $90^{\circ }$, back torsion to the right, left	hip_right_flex
20	lunge, right leg front, knee flexion $90^{\circ }$, elbow flexion $90^{\circ }$, back torsion to the left, right	hip_left_flex

**Table 2:** Joint angles with reference to body planes and positions to which the local body planes are orientated.

Joint Angle	Joint to Body Plane	Angle in Body Plane	Positions to which the Body Planes Are Orientated
neck_flex	frontal	saggital	neck, shoulder_center
neck_latflex	saggital	frontal	shoulder_left, shoulder_right
chest_flex	frontal	saggital	chest, shoulder_center
pelvis_flex	frontal	saggital	hip_center, spine_mid
shoulder_(left, right)_elev	frontal	saggital	shoulder_center, chest
elbow_(left, right)_azim	saggital	transversal	shoulder_center, shoulder_(left, right)
hip_left, right)_flex	frontal	saggital	hip_center, spine_mid

**Table 3:** Differences between the approach used in this paper and WIDAAN.

Property	Approach Paper	Approach WIDAAN
Body Model	individual body dimensions	body dimensions of “the Dortmunder”
Range of Motion	dynamic adaptation through systematic correction, outliers possible	limitation to plausible value ranges, no outliers
Systematic Correction	systematic offsets are corrected using correction function	no correction

**Table 4:** Naming of the joint angles, examined using the approach in this paper, and the corresponding joint angles calculated by WIDAAN.

Approach Paper	Approach WIDAAN	Description
head_inc	Head_Inc_Ang	back and forward tilt of the head
head_latinc	Head_LatInc_Ang	lateral tilt of the head
neck_flex	Neck_Flex_Ang	back and forward tilt of the neck
neck_latflex	Neck_LatFlex_Ang	lateral tilt of the neck
chest_flex		flexion of the spine at chest level
pelvis_flex		flexion of the upper body at pelvis level
shoulder_(left, right)_elev	Shld(L,R)_Elev_Ang	angle between upper arm and upper body
elbow_left, right)_flex	Ellb(L,R)_Flex_Ang	flexion of the elbow
elbow_(left, right)_azim		pointing direction of the forearm to the upper arm
hip_left, right)_flex	Hip(L,R)_Flex_Ang	flexion in the hip to frontal plane
knee_(left, right)_flex	Knee(L,R)_Flex_Ang	flexion in the knee joint
	Thor_Inc_Ang	thorax inclination
	Back_Flex_Ang	back flexion
	Back_Tors_Ang	back torsion
	L5S1_Inc_Ang	angle in the 5th lumbar vertebra and 1st sacral vertebra

**Table 5:** Comparison of our results with WIDAAN results for **tracker configuration 3**.

	Paper		WIDAAN	
**Joint Angle**	**Mean**	**Limits of Agreement**	**Mean**	**Limits of Agreement**
neck flexion	$-0.02^{\circ }$	$-9.64^{\circ }$ and $9.59^{\circ }$	$-0.92^{\circ }$	$-11.35^{\circ }$ and $9.52^{\circ }$
left shoulder elevation	$-0.18^{\circ }$	$-22.76^{\circ }$ and $22.39^{\circ }$	$2.78^{\circ }$	$-7.5^{\circ }$ and $13.05^{\circ }$
left elbow flexion	$0.32^{\circ }$	$-38.74^{\circ }$ and $39.37^{\circ }$	$-24.77^{\circ }$	$-76.89^{\circ }$ and $27.36^{\circ }$
left knee flexion	$-0.29^{\circ }$	$-80.33^{\circ }$ and $79.75^{\circ }$	$-57.18^{\circ }$	$-148.36^{\circ }$ and $34.0^{\circ }$

**Table 6:** Comparison of paper results with WIDAAN results for **tracker configuration 9**.

	Paper		WIDAAN	
**Joint Angle**	**Mean**	**Limits of Agreement**	**Mean**	**Limits of Agreement**
neck flexion	$0.16^{\circ }$	$-26.41^{\circ }$ and $26.73^{\circ }$	$-3.08^{\circ }$	$-22.98^{\circ }$ and $16.82^{\circ }$
left shoulder elevation	$0.08^{\circ }$	$-23.32^{\circ }$ and $23.48^{\circ }$	$2.11^{\circ }$	$-6.23^{\circ }$ and $10.46^{\circ }$
left elbow flexion	$-0.22^{\circ }$	$-40.79^{\circ }$ and $40.36^{\circ }$	$-22.48^{\circ }$	$-68.47^{\circ }$ and $23.51^{\circ }$
left knee flexion	$0.12^{\circ }$	$-23.21^{\circ }$ and $23.45^{\circ }$	$-6.52^{\circ }$	$-41.51^{\circ }$ and $28.46^{\circ }$

**Figure A1:**
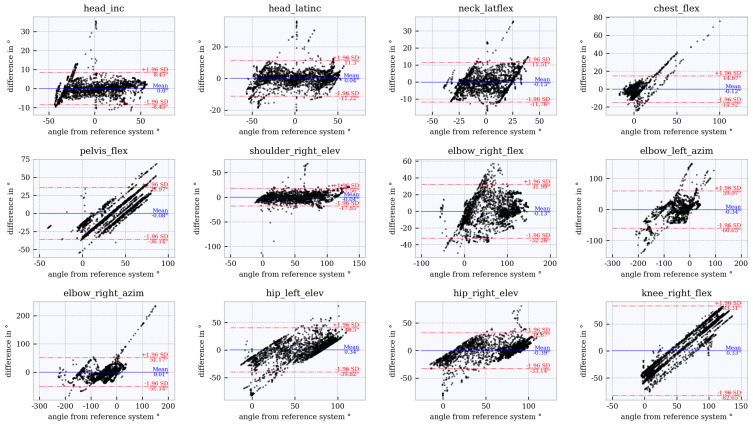
Bland–Altman plot for **tracker configuration 3**; x does not show the average of both systems but the joint angles of the reference system, Qualisys.

**Figure A2:**
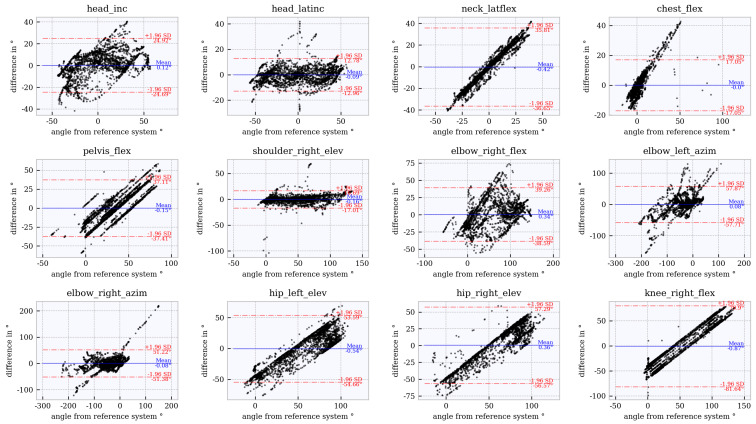
Bland–Altman plot for **tracker configuration 4**; x does not show the average of both systems but the joint angles of the reference system, Qualisys.

**Figure A3:**
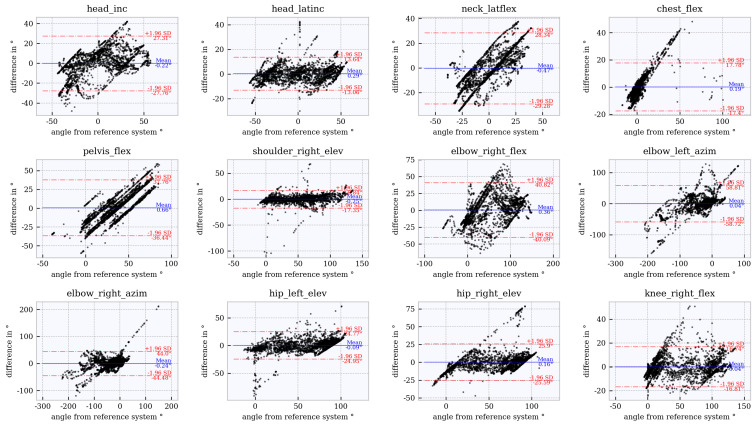
Bland–Altman plot for **tracker configuration 6**; x does not show the average of both systems but the joint angles of the reference system, Qualisys.

**Figure A4:**
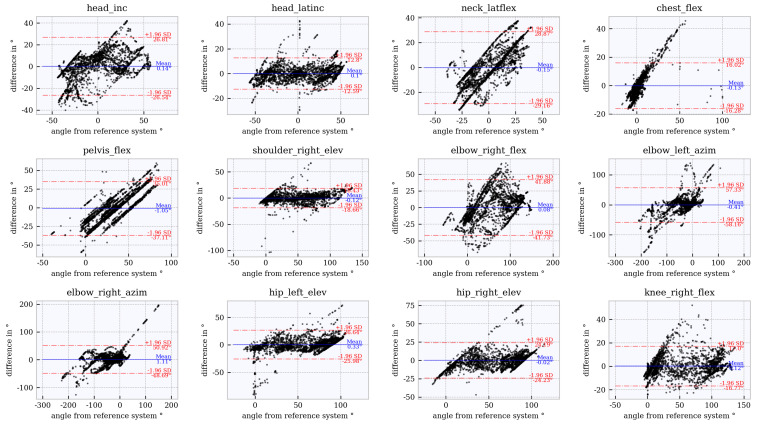
Bland–Altman plot for **tracker configuration 8**; x does not show the average of both systems but the joint angles of the reference system, Qualisys.

**Figure A5:**
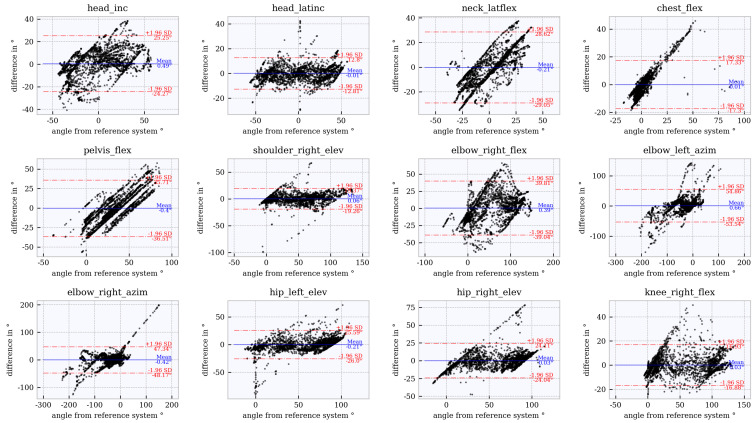
Bland–Altman plot for **tracker configuration 9**; x does not show the average of both systems but the joint angles of the reference system, Qualisys.

## Data Availability

The data presented in this study are available from the corresponding author upon reasonable request.

## Abbreviations

The following abbreviations are used in this manuscript:

CCC	Concordance Correlation Coefficient
HMD	Head Mounted Display
IK	Inverse Kinematic
IPR	Inter-Percentile-Range
LoA	Limits of Agreement
NZM	Neutral Zero Method
REBA	Rapid Entire Body Assessment
RoM	Range of Motion
RULA	Rapid Upper Limb Assessment
VR	Virtual Reality
WIDAAN	Winkel Daten Analyse

## Appendix A

In the appendix, first modified Bland–Altman plots for each tracker configuration (3, 4, 6, 8 and 9) and further joint angles are presented in Figure A1, Figure A2, Figure A3, Figure A4 and Figure A5. Additionally, Bland–Altman plots for further joint angles of configuration 3 and 9 calculated by WIDAAN are presented in Figure A6 and Figure A7. Second, general data statistics are shown in Table A1.

**Table A1:** Data statistics of the analyzed joint angles; 25%, 50% and 75% quartiles, mean value, standard deviation minimum and maximum [${}^{\circ }$].

	Chest Flex	Elbow Left Azim	Elbow Left Flex	Elbow Right Azim	Elbow Right Flex	Head Inc	Head Latinc	Hip Left Elev	Hip Right Elev	Knee Left Flex	Knee Right Flex	Neck Flex	Neck Lat Flex	Pelvis Flex	Shoulder Left Elev	Shoulder Right Elev
**Qualisys**
mean	2.1	−36.9	59.9	−43.6	59.6	0.0	0.5	57.6	57.4	49.8	51.3	2.1	0.7	33.6	42.3	44.4
std	10.7	50.0	48.4	48.6	48.0	26.8	30.9	31.4	32.2	42.4	43.4	21.6	19.0	22.5	36.2	36.7
min	−17.5	−209.1	−62.4	−226.6	−57.2	−43.9	−56.4	−13.2	−15.7	−26.1	−5.6	−41.8	−39.4	−40.6	−15.9	−12.5
25%	−3.2	−56.8	16.4	−64.8	12.0	−24.5	−28.7	31.6	30.3	12.0	10.3	−16.1	−17.0	18.4	5.4	7.1
50%	−0.5	−23.2	61.5	−37.0	60.7	0.1	1.0	68.2	69.0	37.8	39.3	0.4	0.6	32.1	43.0	45.1
75%	3.6	−3.8	101.4	−10.0	101.5	22.4	30.1	84.0	84.6	94.2	97.0	22.7	17.8	51.0	69.8	72.1
max	100.1	111.6	152.9	150.1	149.5	57.2	56.9	109.8	114.7	128.6	135.9	39.6	39.3	86.6	126.8	134.9
**3-tracker**
mean	2.1	−36.9	59.9	−43.6	59.6	0.0	0.5	57.6	57.4	49.8	51.3	2.1	0.7	33.6	42.3	44.4
std	7.3	39.6	43.9	41.6	45.2	26.5	30.4	24.1	27.8	12.8	13.0	21.0	18.0	12.7	34.4	35.5
min	−12.5	−140.5	−64.2	−248.1	−62.8	−40.6	−57.0	−22.1	10.2	5.6	12.6	−36.8	−36.4	−42.1	−4.1	−3.3
25%	−2.2	−57.3	15.5	−54.5	13.3	−26.8	−28.6	36.2	26.8	49.1	41.0	−16.5	−17.8	32.5	6.5	7.1
50%	−1.3	−25.4	56.3	−38.7	58.5	−1.0	0.7	67.1	71.5	53.1	53.2	0.9	0.3	34.0	43.7	45.7
75%	7.7	−5.7	100.5	−12.3	100.5	22.1	31.3	78.8	81.2	58.9	56.7	22.9	20.1	39.8	70.0	72.7
max	28.3	24.5	147.2	37.5	155.1	54.7	56.2	81.9	93.8	69.8	107.4	37.0	34.4	89.4	108.7	114.0
**4-tracker**
mean	2.1	−36.9	59.9	−43.6	59.6	0.0	0.5	57.6	57.4	49.8	51.3	2.1	0.7	33.6	42.3	44.4
std	6.2	40.5	43.1	41.3	43.9	23.5	30.2	15.2	12.6	13.4	11.6	16.1	3.7	12.5	34.5	35.7
min	−15.9	−149.5	−77.9	−219.7	−34.4	−42.8	−50.0	28.4	−2.7	−2.7	−75.0	−29.6	−18.9	−27.4	−3.3	−3.2
25%	−0.8	−61.0	19.8	-59.6	17.7	−18.0	−29.9	45.7	49.4	40.5	41.7	−7.1	−1.6	24.3	6.1	6.7
50%	1.0	−24.7	52.4	−37.1	52.0	−4.7	0.0	51.4	51.5	55.4	48.4	1.7	0.1	35.6	43.5	45.0
75%	3.5	−7.4	102.8	−13.5	102.7	19.3	30.7	69.2	66.0	57.7	64.3	11.4	2.0	38.5	69.4	72.5
max	110.0	48.1	142.6	16.2	152.8	60.2	53.1	98.7	92.5	94.4	107.9	42.9	25.3	81.3	110.7	117.9
**6-tracker**
mean	2.1	−36.9	59.9	−43.6	59.6	0.0	0.5	57.6	57.4	49.8	51.3	2.1	0.7	33.6	42.3	44.4
std	6.2	39.8	43.4	41.2	43.3	23.1	30.1	28.5	29.7	40.8	42.5	16.7	12.0	12.6	34.5	35.7
min	−16.0	−148.1	−71.4	−182.0	−32.6	−43.2	−51.5	−7.7	5.2	−26.7	−2.7	−28.2	−39.0	−29.0	−2.8	−3.1
25%	−0.8	−57.4	18.9	−58.6	18.5	−15.1	−30.6	34.8	24.8	11.4	9.1	−9.1	−8.8	25.1	5.9	6.7
50%	1.0	−29.2	51.7	−37.1	51.8	−6.2	−0.2	69.7	68.7	37.3	38.2	1.9	5.2	35.1	43.3	45.0
75%	3.6	−7.3	103.0	−13.7	102.0	20.7	30.2	81.3	84.0	91.9	97.5	11.0	6.9	38.9	69.4	72.9
max	109.4	43.9	146.8	22.7	154.2	57.9	48.7	90.1	117.4	125.1	134.4	47.7	31.8	84.9	110.7	118.7
**8-tracker**
mean	2.1	−36.9	59.9	−43.6	59.6	0.0	0.5	57.6	57.4	49.8	51.3	2.1	0.7	33.6	42.3	44.4
std	6.2	40.5	43.3	42.0	43.2	23.1	30.1	28.5	29.7	40.8	42.5	16.7	12.0	12.6	34.4	35.4
min	−16.0	−157.9	−71.5	−162.0	−32.5	−43.2	−51.5	−7.5	5.2	−26.7	−2.8	−28.3	−39.0	−29.0	−1.9	−0.4
25%	−0.8	−64.2	19.0	−64.8	18.5	−15.1	−30.6	34.8	24.8	11.4	9.1	−9.0	−8.8	25.1	5.6	7.1
50%	1.0	−26.1	51.7	−32.1	51.9	−6.2	−0.2	69.7	68.8	37.3	38.2	1.8	5.2	35.1	43.5	45.4
75%	3.6	−6.5	102.9	−14.9	102.0	20.7	30.2	81.3	84.1	91.9	97.5	11.1	6.8	39.0	70.4	73.0
max	109.4	36.9	146.5	23.5	154.3	57.9	48.7	90.1	117.4	125.0	134.3	49.0	31.8	84.9	108.2	115.0
**9-tracker**
mean	2.1	−36.9	59.9	−43.6	59.6	0.0	0.5	57.6	57.4	49.8	51.3	2.1	0.7	33.6	42.3	44.4
std	6.4	40.4	43.6	42.4	43.7	23.7	30.2	28.5	29.7	40.7	42.5	16.4	12.0	12.9	34.4	35.4
min	−15.4	−151.5	−70.0	−179.9	−32.8	−43.1	−50.7	−8.6	5.9	−23.3	−2.7	−28.2	−39.3	−15.8	−1.4	−0.8
25%	−1.1	−66.1	18.1	−63.6	17.0	−20.4	−30.6	34.9	24.4	11.5	9.1	−7.1	−8.7	24.8	5.6	7.2
50%	0.7	−24.1	52.7	−33.1	52.7	−2.9	−0.2	69.9	68.6	37.2	38.2	0.2	4.5	34.9	43.5	45.3
75%	4.2	−6.6	103.1	−14.7	101.5	19.4	30.4	81.1	83.7	91.9	97.6	13.0	6.9	39.9	70.4	73.2
max	101.6	56.4	150.7	23.9	153.9	61.0	48.3	90.4	117.6	126.3	133.8	38.0	32.1	67.4	107.5	126.8
